# Marginal Adaptation of New Bioceramic Materials and Mineral Trioxide Aggregate: A Scanning Electron Microscopy Study

**Published:** 2014-03-08

**Authors:** Noushin Shokouhinejad, Mohmmad Hossein Nekoofar, Kazem Ashoftehyazdi, Shohreh Zahraee, Mehrfam Khoshkhounejad

**Affiliations:** a * Dental Research Center, Department of Endodontics, Dental School, Tehran University of Medical Sciences, Tehran, Iran; *; b * Iranian Center for Endodontic Research, Research Institute of Dental Sciences, Shahid Beheshti University of Medical Sciences, Tehran, Iran; *; c * Endodontology Research Group, Dental School, Cardiff University, Heath Park, Cardiff, UK; *; d * Dental School, Tehran University of Medical Sciences, Tehran, Iran*

**Keywords:** Bioceramic, Dental Marginal Adaptation, EndoSequence Root Repair Material, Mineral Trioxide Aggregate, Root Canal Filling Materials, Scanning Electron Microscopy

## Abstract

**Introduction:** This study aimed to compare the marginal adaptation of new bioceramic materials, EndoSequence Root Repair Material (ERRM putty and ERRM paste), to that of mineral trioxide aggregate (MTA) as root-end filling materials.** Materials and Methods:** Thirty-six extracted human single-rooted teeth were prepared and obturated with gutta-percha and AH-26 sealer. The roots were resected 3 mm from the apex. Root-end cavities were then prepared with an ultrasonic retrotip. The specimens were divided into three groups (*n*=12) and filled with MTA, ERRM putty or ERRM paste. Epoxy resin replicas from the resected root-end surfaces and longitudinally sectioned roots were fabricated. The gaps at the material/dentin interface were measured using scanning electron microscope (SEM). Transversal, longitudinal, and overall gap sizes were measured for each specimen. The data were analyzed using the Kruskal-Wallis test. **Results:** In transversal sections, no significant difference was found between MTA, ERRM putty and ERRM paste (*P*=0.31). However, in longitudinal sections, larger gaps were evident between ERRM paste and dentinal walls compared to MTA and ERRM putty (*P*=0.002 and *P*=0.033, respectively). Considering the overall gap size values, the difference between three tested materials was not statistically significant (*P*=0.17). **Conclusion:** Within the limits of this study, the marginal adaptation of ERRM paste and putty was comparable to that of MTA. However, ERRM putty might be more suitable for filling the root-end cavities because of its superior adaptation compared to ERRM paste in longitudinal sections.

## Introduction

An ideal root-end filling material should be biocompatible, nontoxic, dimensionally stable, insoluble in tissue fluids, radiopaque, easily manipulated, and able to seal the root canal system [1, 2]. Mineral trioxide aggregate (MTA) is considered as a suitable root-end filling material as its clinical application has been associated with high clinical success rate and promotion of tissue regeneration [3]. However, it has been criticized for its difficult handling characteristics [4].

In attempt to find materials with similar properties to MTA and improved handling characteristics, new bioceramic materials have been recently introduced. EndoSequence Root Repair Material (ERRM; Brasseler USA, Savannah, GA, USA) has aluminum-free formulation composed of calcium silicates, zirconium oxide, tantalum oxide, calcium phosphate monobasic, and filler agents. It is a premixed product in both moldable putty and low viscosity paste form dispensed from a syringe, which facilitates its application. Since their introduction, bioceramics have been subject of many studies. ERRM has proved to be biocompatible [5-7], antibacterial [8], and able to seal root-end cavities [9]. It has been shown that exposure of ERRM to a synthetic tissue fluid, resulted in precipitation of apatite crystalline structures that was increased over time. This suggests that the materials are bioactive [10].

**Figure 1:**
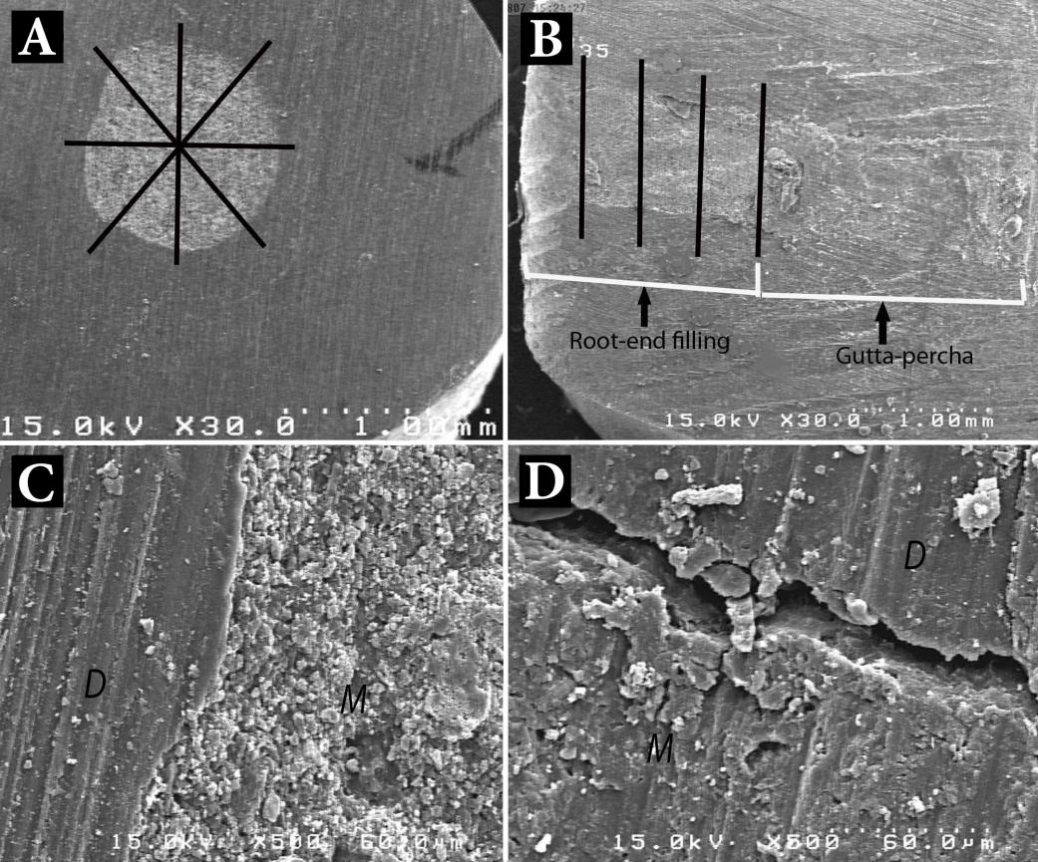
*A)* The perimeter of root-end cavities was divided into eight sections for measurement of gap in transverse sections; *B*) The 3-mm root-end filling material was divided into four sections for measurement of gap in longitudinal sections; *C*) SEM photomicrograph shows no gap between root-end filling material (M) and dentin (D); *D*) A gap is evident at the root-end filling material/dentin interface

Marginal adaptation indirectly reflects the sealing capacity of a root-end filling material; therefore, it has been considered as an important characteristic [11]. Evaluation of marginal adaptation of root-end filling materials by means of scanning electron microscopy (SEM) can provide information regarding their sealing capacity [11, 12]. In several studies, MTA has shown superior marginal adaptation compared with amalgam [13-15], IRM [13, 16], and Super EBA [13, 16]. To date, no information is available regarding the marginal adaptation of endodontic bioceramics. Therefore, this study was designed to compare the marginal adaptation of ERRM putty, ERRM paste, and MTA in resin replicas fabricated from apical surface of the resected root-ends as well as longitudinal root sections defined as transverse and longitudinal replicas, respectively.

## Methods and Materials

A total of thirty-six extracted human single-rooted teeth were selected. The teeth were decoronated to create root samples with standardized length of ~15 mm. The working length was measured by subtracting 1 mm from the lengths recorded when the tip of a #15 K-file (Dentsply, Maillefer, Ballaigues, Switzerland) was visible at the apical foramina. The root canals were prepared using ProTaper rotary system (Dentsply, Maillefer, Ballaigues, Switzerland) to an apical preparation size of #30 (F3). The canals were irrigated between each instrument with 1 mL of 2.5% NaOCl. Then the root canals were dried with paper points and obturated with lateral compaction of gutta-percha (Ariadent, Tehran, Iran) and AH-26 root canal sealer (Dentsply, DeTrey, Konstanz, Germany). The teeth were then stored at 37^º^ C and 100% humidity for 1 week. After that, the apical 3 mm of the roots were resected perpendicular to the long axis of the teeth with a diamond fissure bur (010, Tizkavan, Tehran, Iran) in high speed handpiece under water spray. Three-millimeter deep root-end cavities were prepared with a diamond coated retrotip attached to an ultrasonic unit (Varios 970, NSK, Japan). Specimens were then randomly divided into 3 groups of 12; *Group 1:* White ProRoot MTA (Dentsply, Tulsa Dental, Tulsa, OK, USA) was prepared according to the manufacturer’s instructions. MTA mixture was delivered into the root-end cavities and gently packed with appropriate pluggers and paper points. *Group 2:* ERRM putty was incrementally delivered into the root-end cavities and gently packed with plugger. *Group 3:* Root-end cavities were filled with ERRM paste using the manufacturer-provided preloaded syringe and its delivery tips.

Excessive materials were removed with a plastic instrument. After filling of the root-end cavities, the roots were stored in phosphate-buffered saline (PBS) solution (Dulbecco's Formula Modified, ICN Biochemicals, England), (pH=7.4) at 37^º^ C and incubated for 1 week.

For obtaining transverse resin replicas, impressions were taken from the samples using a polyvinylsiloxane material (Panasil, Kettenbach GmbH & Co. KG, Eschenburg, Germany). A low viscosity epoxy resin (Epoxiran, Tehran, Iran) was mixed with a ratio of 1:1 according to the manufacturer’s instruction and then poured into the impression. Set replicas were then removed. For evaluation of the longitudinal interface between root-end filling materials and walls of the root-end cavity, the roots were ground longitudinally by a diamond bur until the gutta-percha and root-end filling materials were completely exposed. After that, the resin replicas were prepared from longitudinal sections in the manner described for the surface of the resected root-ends. Then replicas were mounted on metallic stubs, gold sputtered and examined under SEM (Vega II XMU, Tescan, Czech Republic). The perimeter of root-end cavities was divided into eight sections (Figure 1A). Then the maximum distance between the root-end filling materials and cavity walls was measured at each section. For longitudinal replicas, the 3-mm root-end filling material was divided into four sections (Figure 1B). In each section, the maximum values observed between the root-end filling materials and both cavity walls were recorded. In order to calculate the gap size value for each transverse and longitudinal replica (Figures 1C and 1D), the average of eight recorded numbers were calculated. Also, the overall maximum gap value for each specimen was calculated by calculating the average of all 16 numbers recorded for both transverse and longitudinal replicas. The mean±standard deviations were calculated.

As the normal distribution of data was rejected, the data were analyzed using the Kruskal-Wallis test. For pair wise comparison of data between and within the groups, the adjusted *P*-values were reported. The significance level was set at 0.05.

## Results

The mean±standard deviations for test materials are shown in the Table 1. The SEM examination of transverse replicas showed no significant difference between MTA, ERRM putty, and ERRM paste (*P*=0.31). However, for longitudinal replicas, the gaps observed in ERRM paste specimens were significantly larger than those seen in MTA and ERRM putty (*P*=0.002 and *P*=0.033, respectively), but there was no significant difference between MTA and ERRM putty (*P*=1.00). Comparing the overall gap size values, the difference between three root-end filling materials was not statistically significant (*P*=0.17).

**Table 1:** Mean (SD) values (µm) for three tested root-end filling materials

**Group**		**Mean (SD)**	**Min.**	**Max.**
**MTA**	**Transverse Section**	6.40 (3.45)	0.00	41.30
	**Longitudinal Section**	2.10 (1.28)	0.00	14.78
	**Total**	4.25 (1.74)	0.00	21.52
**ERRM putty**	**Transverse Section**	2.73 (1.67)	0.00	19.89
	**Longitudinal Section**	2.83 (1.16)	0.00	13.69
	**Total**	2.78 (1.14)	0.00	12.12
**ERRM paste**	**Transverse Section**	0.80 (0.54)	0.00	6.17
	**Longitudinal Section**	8.80 (1.82)^a^	0.78	23.60
	**Total**	4.80 (1.11)	0.39	14.88

a *: significantly different (P>0.05)*

## Discussion

In previous studies on SEM evaluation of marginal adaptation, the interfaces between root-end filling materials and the root canal walls have been evaluated on original teeth samples [17-19], resin replicas [16, 20], or both of them [13, 15]. In the present study, marginal adaptation of MTA, ERRM putty, and ERRM paste was assessed by resin replicas taken from the surface of resected root-ends and longitudinally sectioned roots for more accurate investigation of material adaptation.

It has been stated that preparation of original root specimens before SEM examination might be accompanied with the separation of the material from the root-end cavity walls or crack formation by dehydration of the dental materials and root structure resulting in false positive results [13, 20]. Therefore, the evaluation of resin replicas has been suggested for assessment of marginal gaps to avoid artifacts [21].

Examination of transverse resin replicas showed similar mean gaps in MTA, ERRM putty, and ERRM paste. However, in longitudinal replicas, ERRM paste showed poorer marginal adaptation compared with ERRM putty and MTA. It has been stated that the results of marginal adaptation studies might be impacted by the plane of root section [13]. 

A material may show good adaptation to cavity walls in one plane but not in the entire specimen. However, this study showed similar marginal adaptation in transverse and longitudinal sections of MTA and ERRM putty, but weaker results was found in longitudinal replicas of ERRM paste. The different results between transverse and longitudinal evaluations might be attributed to the physical properties and delivery form of the materials. ERRM putty and MTA mixture can be gently compacted to reduce the voids within the paste as well as the material and walls of the root-end cavity. Low viscosity of ERRM paste makes it impossible to be compacted within the root-end cavity and reduce the possible voids.

The findings of this study showed similar mean gaps for resin replicas of longitudinally sectioned specimens and the surface of resected root-ends filled with MTA. This is not in agreement with the results reported by Torabinejad *et al.* [13] and Badr [15], who showed smaller gap sizes in resin replicas of the apical surface of resected root-ends filled with gray ProRoot MTA compared with those observed in original longitudinal sections. In the present study, resin replicas taken from the longitudinal sections were examined for marginal adaptation but original longitudinal sections were evaluated in the mentioned studies [13, 15]. 

However, in a pilot study, Torabinejad *et al.* showed that the gap sizes in original longitudinal sections were similar to those of replicas taken from longitudinal sections [13]. The controversial results might be attributed to the physicochemical properties and handling characteristics as well as particle size of different formulations of MTA (*i.e.* white ProRoot MTA in the present study and gray ProRoot MTA in the studies by Torabinejad *et al.* [13] and Badr [15]). It has been shown that the particles of white ProRoot MTA were finer than gray ProRoot MTA’s which might influence the physical properties of the material [22, 23].

For comparing the results of studies on marginal adaptation of root-end filling materials, several factors such as design of studies, plane of root sectioning, and methods of gap measurement, should be considered in order to obtain a better comparison between the findings of different studies. Furthermore, different conditions such as type of storage media and the time between placing the root-end filling materials and evaluation of marginal adaptation could affect the results of studies. It has been shown that exposure to blood during setting, had a negative impact on marginal adaptation of MTA compared to materials exposed to synthetic tissue fluid [24]. 

In the present study, the root-end filling materials were exposed to PBS to partially simulate the *in vivo* conditions in which MTA is used [25, 26]. In some studies, it has been stated that the roots were stored in 100% humidity and the type of storage media have not been clarified [15, 27]. In the other studies, the roots were stored in water [16] or wrapped in moist gauze and stored in 100% humidity [14]. 

The interaction of biomaterials like MTA with storage media is an important issue that should be considered in studies on the properties of biomaterials. Several studies showed that the interaction of MTA with a phosphate-containing solution such as PBS resulted in the formation of apatite crystals [10, 25, 28]. Calcium ions released by MTA, react with phosphate in PBS, resulting in the formation of hydroxyapatite [25] or carbonated apatite [28, 29]. This phenomenon has been proposed as a mechanism of decreasing leakage [28]. 

Although the precipitation of apatite appears to begin within the first hours of immersion in PBS, aggregation of greater amounts of apatite crystals and formation of interfacial layer at dentin-material interface has been shown in long terms [10, 25, 28]. Therefore, one-week storage in this study was insufficient to make a significant difference between transverse and longitudinal sections. If the specimens were incubated for a longer time, it might affect the marginal adaptation of transverse sections more than longitudinal sections.

The present study showed no significant difference between the overall gap size values for three examined materials. From the similar results obtained for MTA and ERRM regarding the sealing ability [9], biocompatibility [5-7], and bioactivity [10], it would be expected that the marginal adaptation of ERRM is comparable to that of MTA. However, it is worth mentioning that further clinical studies are needed for confirmation of these results.

## Conclusion

Under the conditions of this *in vitro* study, the marginal adaptation of new bioceramic materials, ERRM paste/putty was comparable to that of MTA. However, it might be better to use ERRM putty or MTA in filling of the root-end cavities because of their superior adaptation compared to ERRM paste in longitudinal sections.
